# The MemProtMD database: a resource for membrane-embedded protein structures and their lipid interactions

**DOI:** 10.1093/nar/gky1047

**Published:** 2018-11-12

**Authors:** Thomas D Newport, Mark S P Sansom, Phillip J Stansfeld

**Affiliations:** Department of Biochemistry, University of Oxford, South Parks Road, Oxford, OX1 3QU, UK

## Abstract

Integral membrane proteins fulfil important roles in many crucial biological processes, including cell signalling, molecular transport and bioenergetic processes. Advancements in experimental techniques are revealing high resolution structures for an increasing number of membrane proteins. Yet, these structures are rarely resolved in complex with membrane lipids. In 2015, the MemProtMD pipeline was developed to allow the automated lipid bilayer assembly around new membrane protein structures, released from the Protein Data Bank (PDB). To make these data available to the scientific community, a web database (http://memprotmd.bioch.ox.ac.uk) has been developed. Simulations and the results of subsequent analysis can be viewed using a web browser, including interactive 3D visualizations of the assembled bilayer and 2D visualizations of lipid contact data and membrane protein topology. In addition, ensemble analyses are performed to detail conserved lipid interaction information across proteins, families and for the entire database of 3506 PDB entries. Proteins may be searched using keywords, PDB or Uniprot identifier, or browsed using classification systems, such as Pfam, Gene Ontology annotation, mpstruc or the Transporter Classification Database. All files required to run further molecular simulations of proteins in the database are provided.

## INTRODUCTION

There are now ∼3500 structures of over 1000 unique integral membrane proteins deposited in the Protein Data Bank (PDB) (1,2). Membrane proteins are of considerable biomedical interest, constituting ∼25% of published genomes (3) and 50% of current drug targets (4). A near-exponential increase in the number of published membrane protein structures (5) looks set to be sustained through continuous improvements to detergent solubilization and crystallization protocols, such as Lipidic Cubic Phase (6), HiLiDe (7) and MemGold (8). Meanwhile, the enhanced resolution and variety of structures solved by Cryo-Electron Microscopy (9) opens up a wealth of new possibilities.

With rapid growth in the number of protein structures available, several database systems have been developed in order to organize and annotate these structures in a biologically meaningful way. The PDB is a universal repository for all experimentally derived membrane protein structures, holding a single accession for each deposited structure. Structures deposited in the PDB may be linked to one or more UniProt (10) protein sequence, which in turn can be grouped into larger Pfam (11) families. In addition the Gene Ontology (GO) project (12) defines a standardized way to further annotate proteins with attributes ranging from functional notes to cellular location. Several specialized classification systems also exist to classify membrane proteins; Membrane Proteins of Known Structure (mpstruc; http://blanco.biomol.uci.edu/mpstruc/) (13) places membrane protein structures into a hand-curated tree, whilst the Transporter Classification DataBase (TCDB; http://www.tcdb.org) (14) provides an automatically generated tree looking specifically at transporters and channels. The GPCRdb provides comprehensive data, diagrams and web tools for G protein-coupled receptors (GPCRs; http://gpcrdb.org) (15). The membrane protein database (MPDB; http://www.mpdb.tcd.ie) details the conditions and additives used to experimentally solve membrane protein structures (16).

Membrane proteins are embedded in a lipid bilayer, which has significant influence on both the structure and function of the protein. Whilst it is not uncommon to be able to identify a few lipids when determining a membrane protein structure (17), most lipid components of the membrane are highly dynamic and rapidly exchanged (18), so are not readily resolved by current structure determination techniques.

Methods such as Positioning of Proteins in Membrane (PPM) (19), TMDET (20) and Memembed (21) can use the distribution of surface amino acid residues to infer both the tilt of the protein and the thickness of the lipid bilayer, modelling the bilayer as a slab bounded by two planes. PPM is used in the Orientations of Proteins in Membranes (OPM; https://opm.phar.umich.edu) database, while TMDET is applied to entries in the Protein Data Bank of Transmembrane Proteins (PDBTM; http://pdbtm.enzim.hu). Although these methods can accurately predict the location of transmembrane (TM) domains, they lack explicit lipids and so are unable to capture specific interactions between individual lipid molecules and sites on the surface of the protein. Highly specific protein–lipid interactions can modulate protein function, either directly (22) or by regulating protein–protein interactions such as oligomerization (23,24). These lipid-binding sites yield potential targets for novel allosteric modulatory drugs from within the membrane, e.g. AZ3451 binding to the PAR2 receptor (25). In other instances, interactions between the protein and specific lipid molecules result in chemical modification of the lipid (26), or the translocation of a lipid molecule between leaflets of the bilayer (27). In several cases, the structure of the protein may induce local deformations of the membrane, thinning or thickening the membrane or inducing curvature (28).

Molecular Dynamics (MD) simulations permit detailed models of protein interactions within biological membranes, characterizing the interactions of a protein with explicit lipid molecules. MD simulations are typically performed on one of two scales: atomistic MD simulations consider each atom in the simulated system as a single particle, producing more accurate simulations, whilst Coarse-Grained (CG) MD simulations treat 3–4 heavy atoms as a single particle (29), reducing the complexity of the system and allowing for longer or larger simulations. Multi-scale approaches simulate a system using both CG and atomistic representations, combining the strengths of both approaches (30,31).

Several methods exist to reconstitute lipid membranes using MD simulations. The CHARMM-GUI (32) lipid-builder tool can either be used to insert a protein structure into a pre-equilibrated bilayer, or generate a new bilayer by packing lipids around a protein structure, whilst INSANE (33) initially builds a CG membrane on a planar grid around the protein. The MemProtMD pipeline (34) instead uses a CG Self-Assembly (CGSA) approach, whereby lipids are initially placed and oriented randomly around the protein, and a short CG simulation is performed, during which a lipid bilayer forms spontaneously (35). The assembled system can then be simulated further in either CG or atomistic representation (36).

Here, we present the MemProtMD database, an online resource which stores and analyses the results of simulations of a complete set of integral membrane protein structures in explicit dipalmitoylphosphatidylcholine bilayers, set up using the MemProtMD pipeline (34). This database builds on earlier concepts first formulated for the CGDB (37). Protein–lipid interactions, membrane deformations, ensemble analyses and bulk membrane properties from the MD simulations are assessed and stored in a database. These data are combined with annotations from external databases to classify proteins and identify conserved protein–lipid interactions across both proteins and families. The database can be browsed using a freely accessible web application (http://memprotmd.bioch.ox.ac.uk), and results of analyses, as well as files and instructions required to perform further simulations can be downloaded.

## MATERIALS AND METHODS

### Implementation

The initial CGSA simulation setup is performed using the MemProtMD pipeline (34). In brief, integral membrane proteins are identified from the PDB based on an Octopus prediction of surface-assessible α-helical TM domains (38). Potential β-barrel membrane proteins are identified based on the number, length, accessibility and hydrophobicity of their β-strands. Where available, the biological unit in the PDB is prepared for the oligomeric state of the simulated protein. In all instances non-protein atoms are removed from the PDB entry prior to simulation. MD simulations are performed using GROMACS 5.1.4 (39) and the MARTINI 2.2 forcefield (40). Completed simulations are then converted to atomistic representation using CG2AT (30). Analysis of completed simulations is performed using Python, MDAnalysis (41) and our *in house* mpm-tools, which includes bindings for MUSCLE (42) and a python adaptor for VMD (43), used to render static images. Two dimensional visualizations of data are performed using D3.js. Three dimensional protein visualization uses PV. The database is stored using MongoDB. The web server uses NodeJS and Express to serve a frontend application built on ReactJS and Redux. Server application deployment is performed using Docker Compose.

## RESULTS

### The MemProtMD database and web server contents

**Figure 1.**
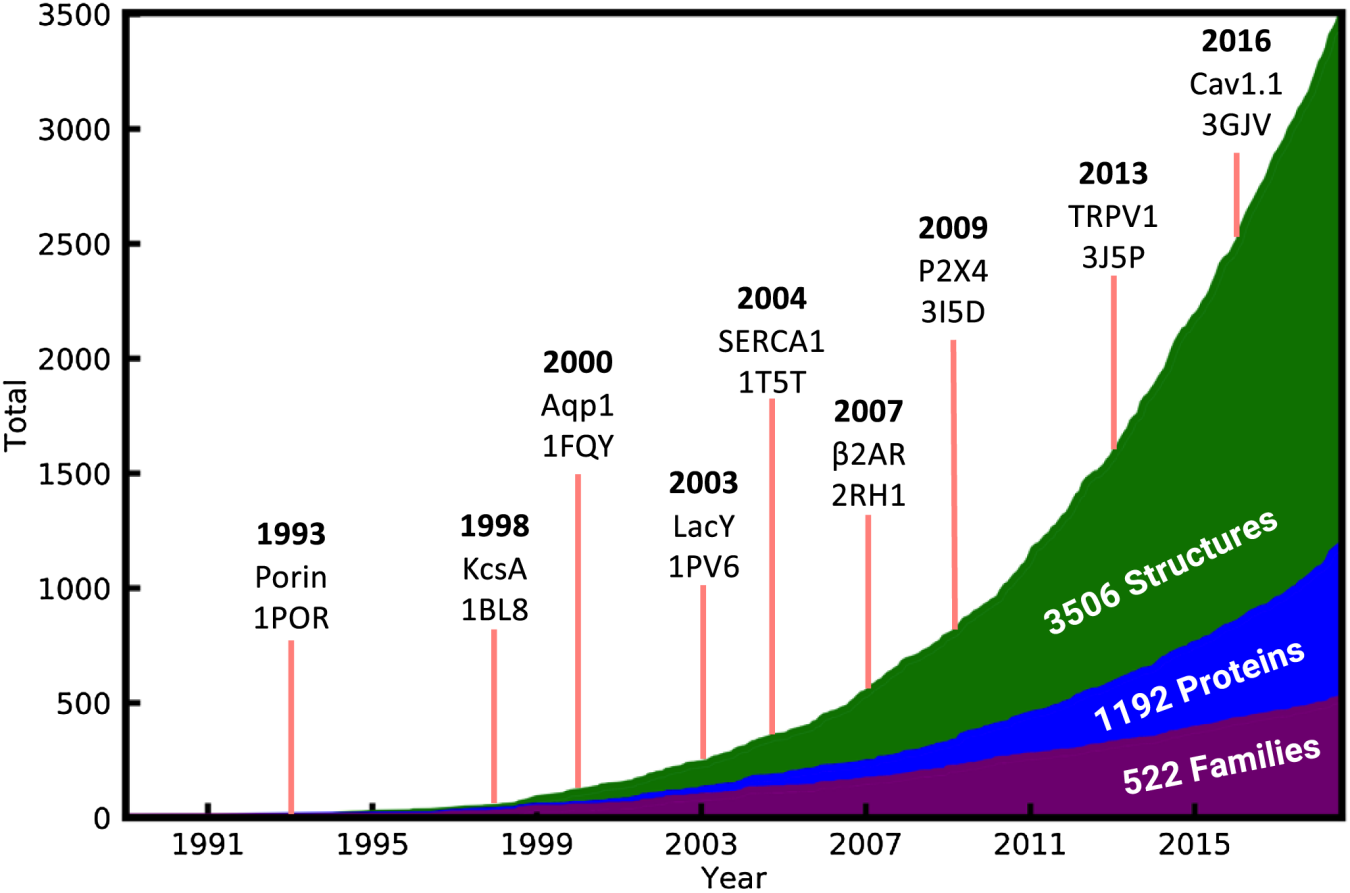
The number of unique PDB, UniProt and Pfam accessions represented in the MemProtMD database over time. A selection of landmark structures are indicated by red lines, denoting their PDB release date and include the first Porin (44), K^+^ channel (45), aquaporin (46), solute transporter (47), Sec translocon (48), adrenergic receptor (49), glutamate receptor (50), TRP channel (51) and voltage-gated Ca^2+^ channel (52). The total number of PDB structures, UniProt proteins and Pfam families are shown.

At time of writing, the MemProtMD database holds 3506 CGMD simulations of intrinsic membrane proteins inserted into phospholipid bilayers, each based on a single structure deposited in the PDB. Represented within these are TM proteins, which correspond to 1192 unique UniProt proteins and 522 Pfam families. The cumulative number of intrinsic membrane protein structures deposited in the PDB continues to rise at an almost exponential rate, as shown in Figure 1. This figure contains a timeline of a selection of key membrane protein structures (44–52). If the same trend is followed, we expect that in 10 years there will be ∼10 500 membrane protein PDB structures, ∼3500 unique proteins and ∼1200 Pfam entries. The most up-to-date values can be found at http://memprotmd.bioch.ox.ac.uk/stats.

**Figure 2.**
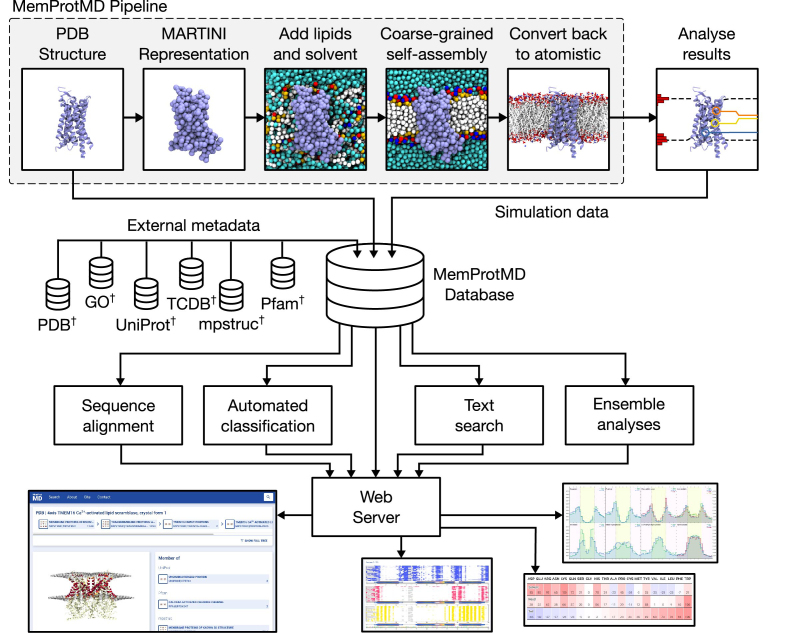
Overview of the MemProtMD pipeline and database. Membrane protein structures deposited in the PDB are first converted to MARTINI representation. Lipids and solvent are then added with random orientations and a 1 μs self-assembly simulation is performed. The simulation is converted back to atomistic representation using CG2AT and several analyses are performed. Analysis results, as well as metadata derived from the original PDB structure and metadata downloaded from a range of databases are deposited into the MemProtMD database, which is then used to perform multiple sequence alignments, automatically classify membrane proteins, perform text searches and perform ensemble analyses.

As part of our workflow, intrinsic membrane proteins are automatically identified and downloaded on a weekly basis as they are released from the PDB in Europe (2), using the MemProtMD pipeline (34). At present, this averages ∼8 structures per week. In 10 years’ time we expect this to be closer to 20 structures per week. We expect that due to further methodological advances, this is likely to be a conservative estimate. Upon identification, the coordinates are converted to a CG representation for a self-assembly simulation with phospholipids, water and ions (35). This 1 μs MD simulation is performed and the final frame converted back to atomistic representation using CG2AT (30). This process is shown schematically in Figure 2. If the protein successfully inserts into a lipid bilayer during the simulation, a set of analyses are performed to identify contacts between lipid molecules and the protein surface, as well as characterize the surface of each leaflet of the lipid bilayer. These data are then deposited in the MemProtMD database along with CG and atomistic snapshots of the final simulation frame.

Analysis of specific amino acid residues in structures sharing UniProt or Pfam accessions is then aggregated either according to the aligned amino acid sequence of the protein, or the position of the residue relative to the annular lipids of the simulated bilayer. The results of simulations and subsequent analysis can be visualized and downloaded using the MemProtMD web server, available at http://memprotmd.bioch.ox.ac.uk.

### Simulation analysis

**Figure 3.**
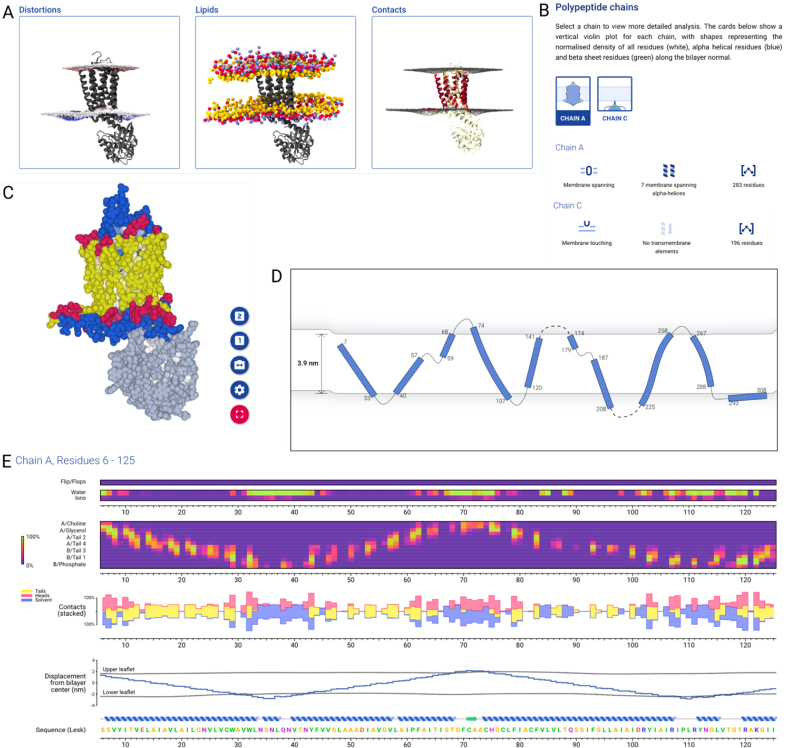
Online analysis and visualization of a simulation of the A_2A_ adenosine receptor. (**A**) Pre-rendered images available to view and download showing (from left to right) protein with membrane surface shaded to show thinning (red) and thickening (blue), protein with CG lipid head-group beads (yellow: glycerol, red: phosphate, blue: choline) and protein surface shaded according to contacts with lipid head-groups. (**B**) Chain-by-chain overview of the protein, showing violin plot of protein density along membrane normal, classification of chain membrane interactions (‘Membrane spanning’), topology summary (‘7 membrane spanning α helices’) and residue count. (**C**) Online 3D protein view showing protein in sphere representation coloured by membrane contact type (yellow: acyl tails, red: lipid head-groups, blue: solvent). The selected chain (Chain A) is shown in full colour, whilst other parts of the protein are desaturated. (**D**) Topology viewer showing tilt and curvature of TM helices. Non-continuous loops are shown as dashed lines. The membrane thickness and degree of protein-induced deformation is shown. (**E**) Sequence viewer showing several metrics along the amino acid sequence: (from top to bottom) contacts with lipids flipping from one leaflet to another; contacts with water and ions; contacts with lipids, broken down by chemical group; stacked plot of contacts with acyl tails, head-groups and solvent; position of the protein and local membrane thickness; protein secondary structure and amino acid sequence.

Completed simulations pass through an analysis pipeline which performs several assessments to characterize the interactions between lipid bilayer and membrane protein. First, the upper and lower leaflets are identified, as well as any movement of lipid molecules between leaflets. A radial distribution function of lipid head groups around the protein is then calculated and used to determine the shape of annular shells of lipids around the protein. The surfaces formed by each leaflet are then characterized, as well as distortions in these caused by the protein (Figure 3A). Contacts between the protein and different parts of the surrounding lipids and solvents are then calculated, and a set of static images is rendered (Figure 3).

The results of these analyses can be searched for and visualized in a modern web browser using the MemProtMD server (Supplementary Figure S1). An overview for each protein shows metadata derived from external databases, as well as links to download files to run new simulations (Supplementary Figure S2). Pre-rendered images can be browsed (Figure 3A), and a summary of the topology of protein chains can be used to select an individual chain (Figure 3B). This selection is reflected in an online 3D viewer (Figure 3C), which can be configured to show a snapshot of the protein and membrane lipids, coloured by metrics derived from simulation analyses and selectable by the user. When a protein chain is selected, a 2D topology viewer shows a simplified view of each secondary structure element, using a B-spline approximation which preserve bends and kinks (Figure 3D). Unlike other topology viewers, this also captures the thickness changes of the explicit membrane around the protein.

A sequence view of the selected chain (Figure 3E) shows metrics mapped to the amino acid sequence of the protein. Two coloured series at the top identify amino acid residues which contact leaflets flipping between two bilayers and amino acids which contact water and ions. Contacts with each CG bead of membrane lipids are then shown for each amino acid residue, starting with the choline bead of the upper leaflet, progressing through phosphate beads, glycerol beads and the four beads representing acyl tails of the lipid. Below this is a plot showing contacts with three larger groups of beads; lipid head-group beads, lipid acyl tail beads and solvent beads, represented as stacked bars. The displacement of each protein residue from the bilayer centre is calculated, as well as the displacement of the closest point on the surface of each membrane leaflet. Using this calculation, it is possible to display bilayer thickness and distortions along the amino acid sequence, identifying sets of residues which may be involved in distorting the membrane.

### Ensemble analyses

**Figure 4.**
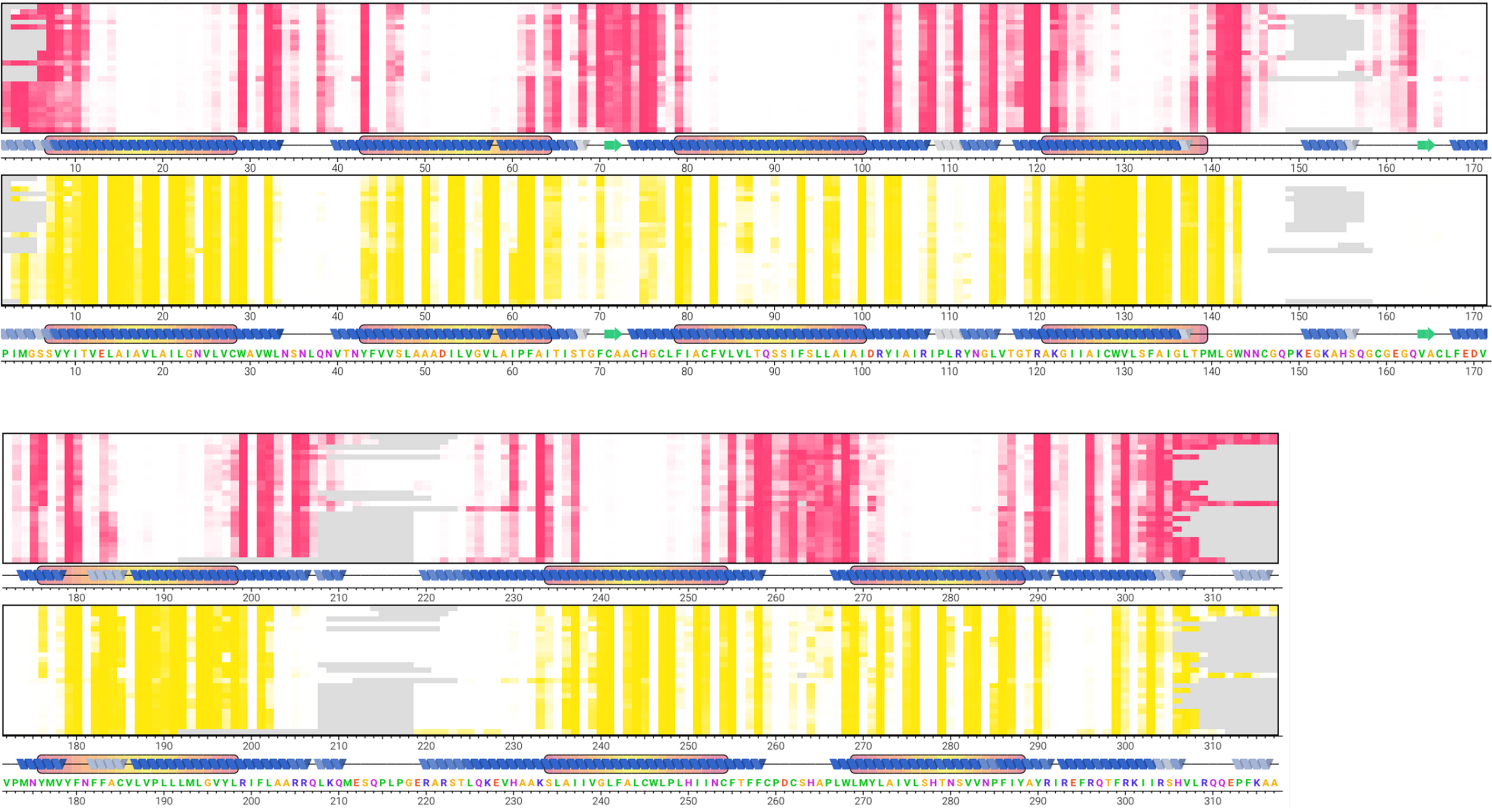
Online sequence view for 25 simulations of the UniProt AA2AR_HUMAN entry (Uniprot id: P29274) contacts with lipid head-groups (red) and lipid acyl tails (yellow) are shown arranged along the protein amino acid sequence, with each row representing a single simulated structure. A consensus secondary structure is shown at the bottom of each row for each PDB file, shaded darker where secondary structure is more highly conserved within the structures. TM domains are shown by a box around the secondary structure, shaded according to mean depth within the membrane, from red (shallow) to yellow (deep), where a certain residue was not present in a structure the cell is shaded grey.

The 3506 membrane protein simulations, shown in Supplementary Figure 3, enable single-entry, collective and large-scale evaluation of general membrane protein properties. For a given UniProt identifier with multiple PDB entries, per-residue analyses of the aggregated simulations may be viewed along the protein sequence, shown here for the multiple structures of the human A_2A_ adenosine receptor (Figure 4). This reveals a regular and highly conserved pattern of membrane head group and tail contacts, in addition to solvent exposed residues. As part of this analysis consensus, secondary structure elements and TM domains of the protein are shown. This methodology is also extended to each Pfam family by aligning all related UniProt sequences using MUSCLE (42). By incorporating the lipid contact information, an equivalent sequence alignment may then be produced for each of the 522 Pfam families in MemProtMD, as we performed previously, in a non-automated manner, for the Aquaporin family (53). This reveals a highly conserved pattern of lipid contacts across all GPCRs within the 7TM receptor Pfam (PF00001), which currently includes 222 protein structures (Supplementary Figure S4). Other exemplar Pfam entries include that of the POT family (PF00854), the Sodium:neurotransmitter symporter family (PF00209) and an updated version of the Aquaporin family, now with 51 entries, using the Major intrinsic protein Pfam (PF00230).

Using amalgamated simulation data from every single protein in the MemProtMD database, further light can be shed on the distribution of amino acids within lipid bilayers. For each residue type, the unbiased probability of a contact between that residue and either lipid head-groups, lipid acyl tails or solvent is calculated (Supplementary Figure S5A). The probabilities are used to construct a normalized scale, with glycine assigned a value of 0 and the maximum probability being 100. Due to our explicit lipid methodology, we can unpack these data further, capturing the specific portion of the lipid that interacts preferentially with each amino acid residue, as we previously performed for aquaporins (53). As we observed for the aquaporin dataset, the two basic residues, arginine and lysine, preferentially interact with the phosphate group, tyrosine and tryptophan form the majority of interactions with the glycerol region of the lipid, while phenylalanine, leucine, isoleucine, tryptophan and valine most commonly form contacts with the terminal lipid tails. (Supplementary Figure S5B). These data dynamically updates on a weekly basis, with the latest data found at http://memprotmd.bioch.ox.ac.uk/stats.

Also displayed on this page is the mean distribution of surface amino acid residues for all simulations. This is visualized along the bilayer normal (Supplementary Figure S6). Pore inner residues are discounted from the analysis, and each protein residue is assigned a coordinate for displacement along the membrane normal, normalized to account for variations in membrane thicknesses. Residue types with similar distributions are clustered together. Histograms for each amino acid are normalized to sum to 1. The data may be dynamically viewed based on the absolute residue distribution or normalized to the relative number of the amino acid species. As observed for a smaller dataset (54), hydrophobic residues isoleucine, leucine, valine and phenylalanine are found in the membrane core, the aromatic residues tryptophan and tyrosine are found at the membrane–solvent interface, near the more solvent exposed arginine and lysine.

Global analysis also illustrates a clear difference in the membrane thickness (phosphate centroid to phosphate centroid) proximal to the protein, between bilayers containing α-helical proteins, typically ∼37 Å in thickness, and membranes of β-barrel proteins, typically ∼33 Å in thickness (Supplementary Figure S7). This is likely due to the reduced hydrophobic thickness of bacterial outer membranes, in which most of the β-barrel membrane proteins reside.

### Integration with other databases

Protein structures are initially identified by their PDB deposition. In MemProtMD, the links to the PDB are maintained, with PDB metadata used to populate the database. Structures are grouped according to their constituent proteins, as defined by UniProt accessions, and according to their family, defined by Pfam accessions. A grouping of proteins is also created from each GO annotation. Each node defined by the TCDB and mpstruc trees is also used to group proteins, using the classifications from the external databases. A page showing all simulations in the defined group, as well as statistics for that group (where the group has more than 50 structures) can be viewed online. We have also configured our own ‘mpm’ annotation scheme, with links to groupings of GPCRs, Ion channels, Outer membrane proteins, Aquaporins and ATP Binding Cassette Transporters, which are shown on the home page of the website.

### Search and classification tools

The MemProtMD database automatically classifies proteins based on a variety of criteria. These may include keywords, references to accessions in external databases or metrics derived from simulations of the protein. These classifications are applied automatically and are then refined manually as needed. An example of the automated classification is shown in Supplementary Figure S2 for the A_2A_ GPCR protein. The MemProtMD database can also be searched using the MemProtMD web server using a text-based search against keywords, titles, accessions and descriptions of entries in external databases. Results are returned, ordered by relevance and may include individual simulations or collections comprising of several simulations sharing a particular reference to an external database.

## CONCLUSION AND OUTLOOK

Here we have described the MemProtMD database, which houses automated MD simulations and analyses for membrane protein structures embedded in explicit lipid membranes. In addition to displaying data for individual PDB entries, ensemble analyses have also been performed to characterize lipid interactions with multiple Uniprot entries and Pfam families. The database is designed for sustainability with automated weekly updates based on the latest release of membrane protein structures. Going forward we will expand the database to incorporate a diverse array a membrane lipid species (33), selected based on the nature of the membrane, e.g. bacterial or eukaryotic, endosomal or plasma membrane. This will enable the identification of specific lipid binding sites within the annular shell.

## DATA AVAILABILITY

The MemProtMD database can be accessed through the web server at http://memprotmd.bioch.ox.ac.uk. Regular updates of new entries are provided from the Twitter account @memprotmd.

## Supplementary Material

Supplementary Data
