# Supplementary material for: The MemProtMD database: a resource for membrane-embedded protein structures and their lipid interactions
Source: Nucleic Acids Res. 2018 Nov 12;47(Database issue):D390–7. doi: 10.1093/nar/gky1047 (PMC6324062; doi:10.1093/nar/gky1047)
Supplement: Supplementary Data [file gky1047_supplemental_files.docx]

# Supplementary Figures

**Figure 1: Home page of the MemProtMD web application**

**Figure 2: Online structure reference view for an A_2A_ Receptor**

**Figure 3: A montage of all MemProtMD entries**

**Figure 4: Multiple sequence alignment view the 7-TM Receptor (GPCR) Pfam**

**Figure 5: Per amino-acid scales of lipid-contact frequency**

**Figure 6: Histograms of amino acid frequency**

**Figure 7: Structure, topology and membrane Interactions for** β**-barrel proteins**

**
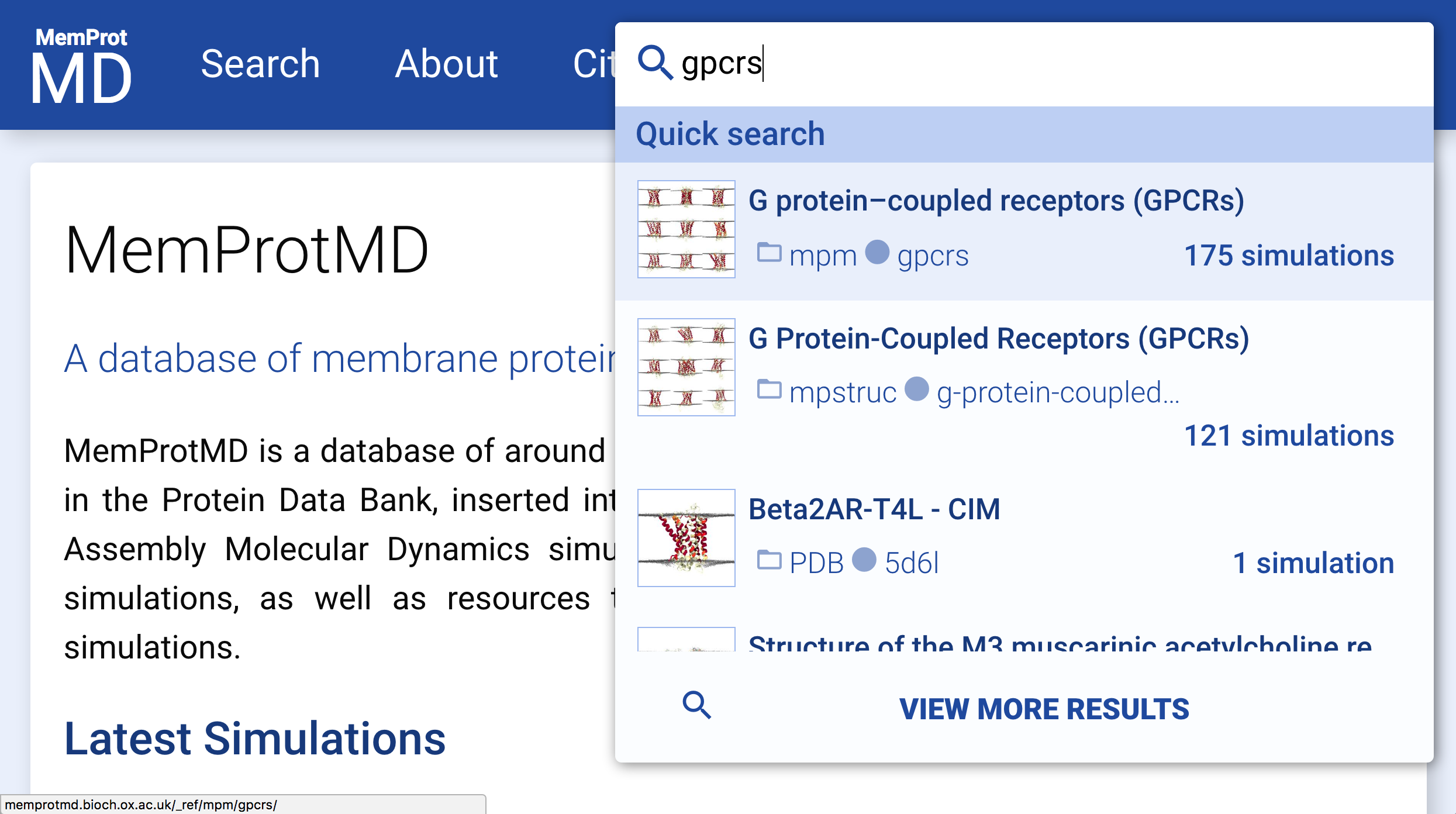
**

**Supplementary Figure 1: Home page of the MemProtMD web application.** The quick search utility is shown performing a search for simulations of GPCR structures.


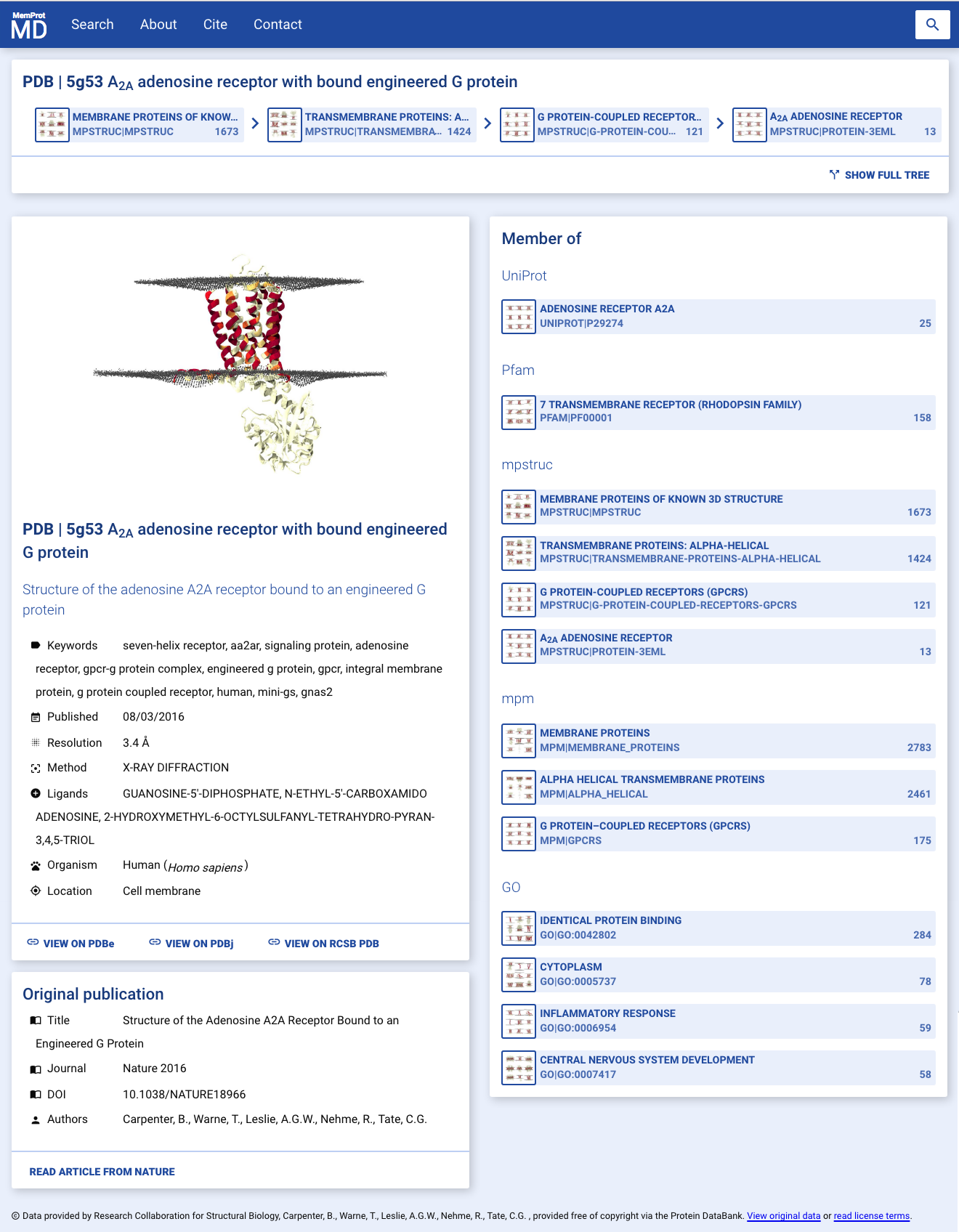


**Supplementary Figure 2: Online structure reference view for an A_2A_ Receptor.** An overview of the header associated with all PDB entries, showing structural details, publication information, and links to similar Uniprot, Pfam, mpstruc, GO and mpm grouped entries (PDB id: 5G53).

**
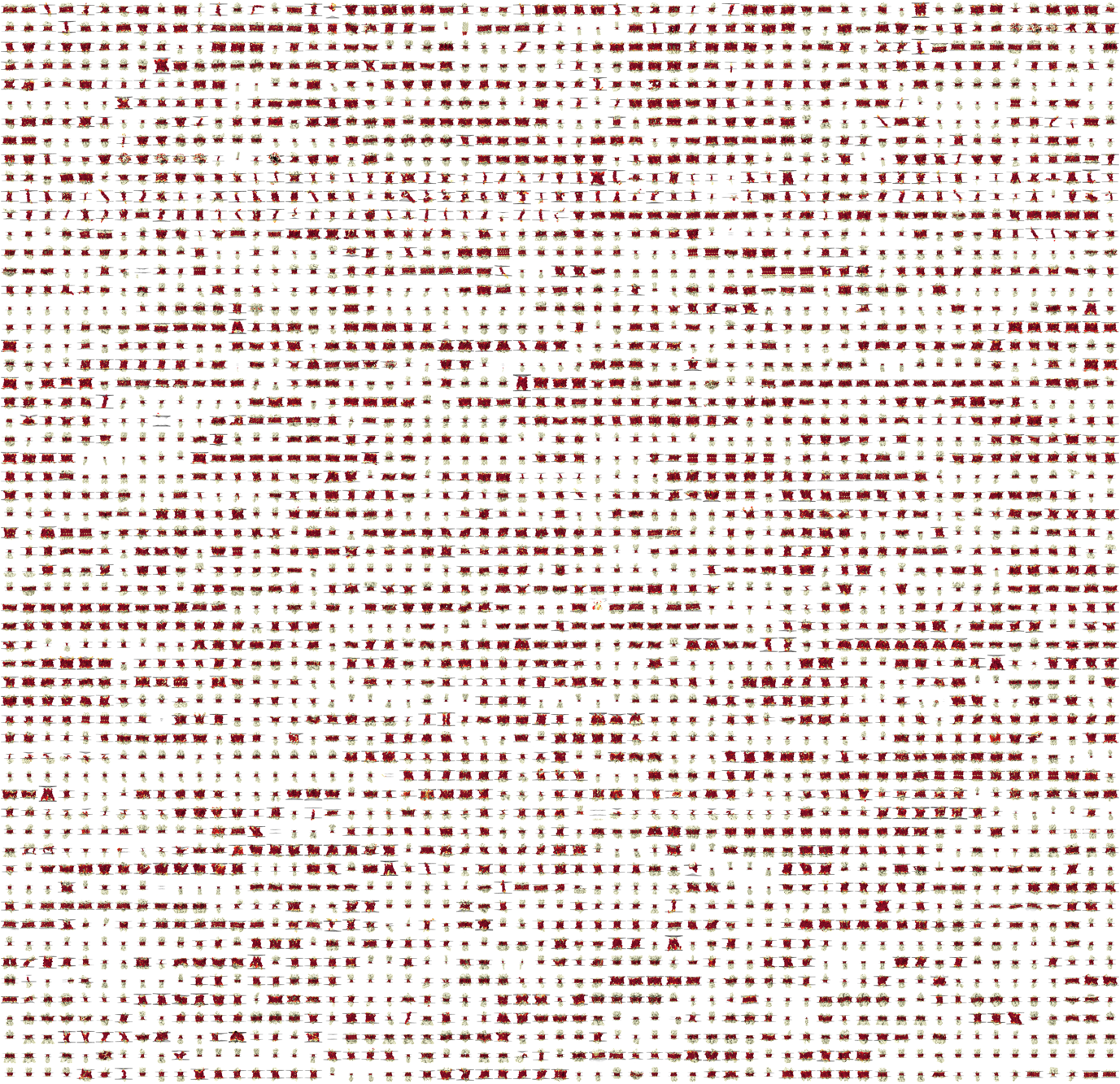
**

**Supplementary Figure 3: A montage of all MemProtMD entries.** Proteins are shown in surface representation coloured red by lipid contacts. Bilayer headgroups are shown in black dots.

**Supplementary Figure 4: Online multiple sequence alignment for the 222 simulations of proteins in the 7-TM Receptor (GPCR) Pfam.** Contacts with lipid head-groups (red) and lipid acyl tails (yellow) are shown arranged along a multi-sequence alignment, with each row representing a single simulated structure. A consensus secondary structure is shown at the bottom of each row, shaded darker where secondary structure is more highly conserved. TM domains are shown by a box around the secondary structure, shaded according to mean depth within the membrane, from red (shallow) to yellow (deep). Where a certain residue was not present in a structure, or a gap exists in the multi-sequence alignment, the cell is shaded grey.

**
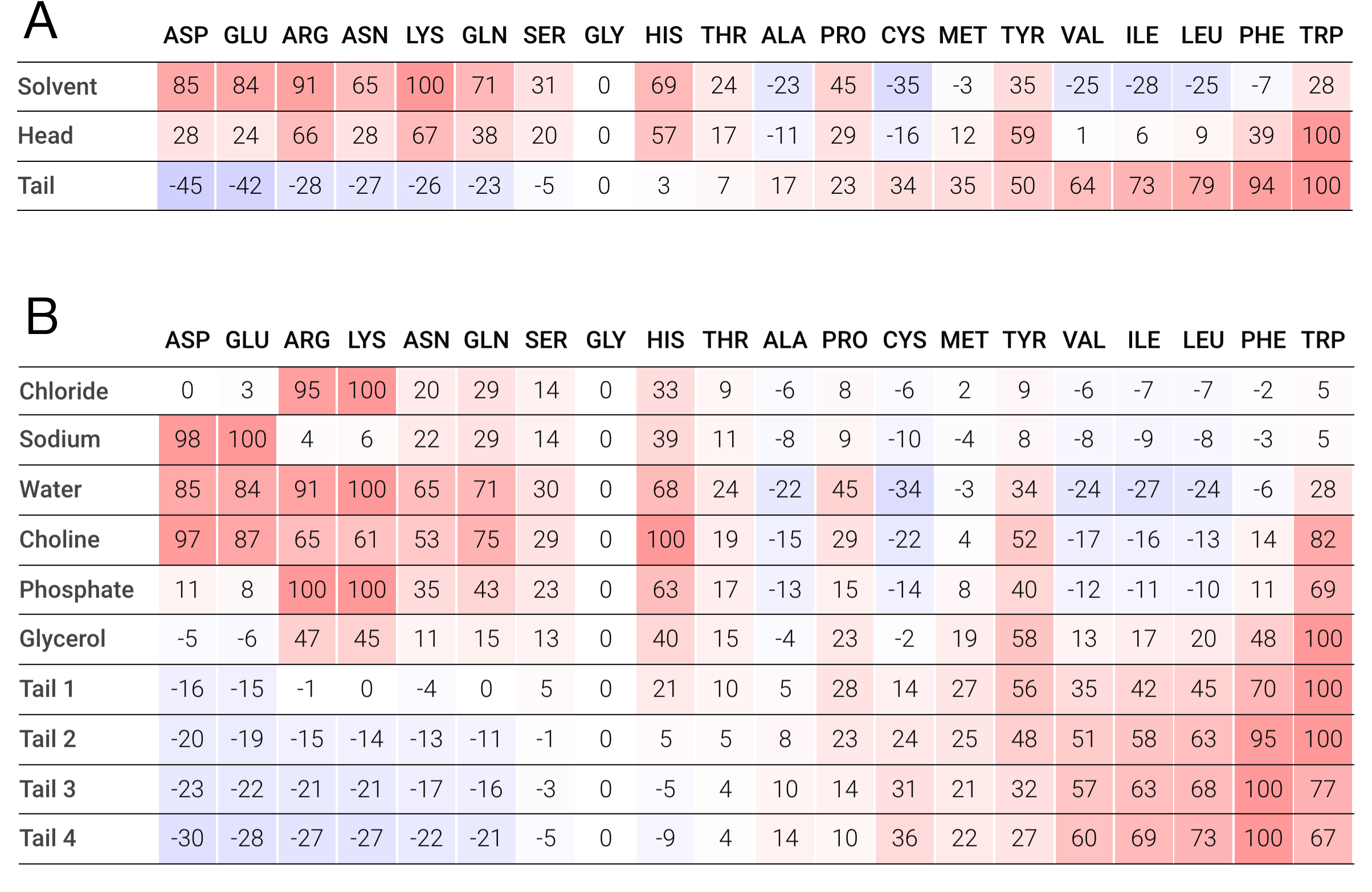
**

**Supplementary Figure 5: Per amino-acid scales of contact frequency with different membrane components.** Normalised such that the value of Glycine is 0 and the maximum value is 100. **A)** Contacts grouped by solvent, lipid head-groups and lipid acyl tails **B)** contacts grouped by chemical group, corresponding to MARTINI beads used for simulation.

**
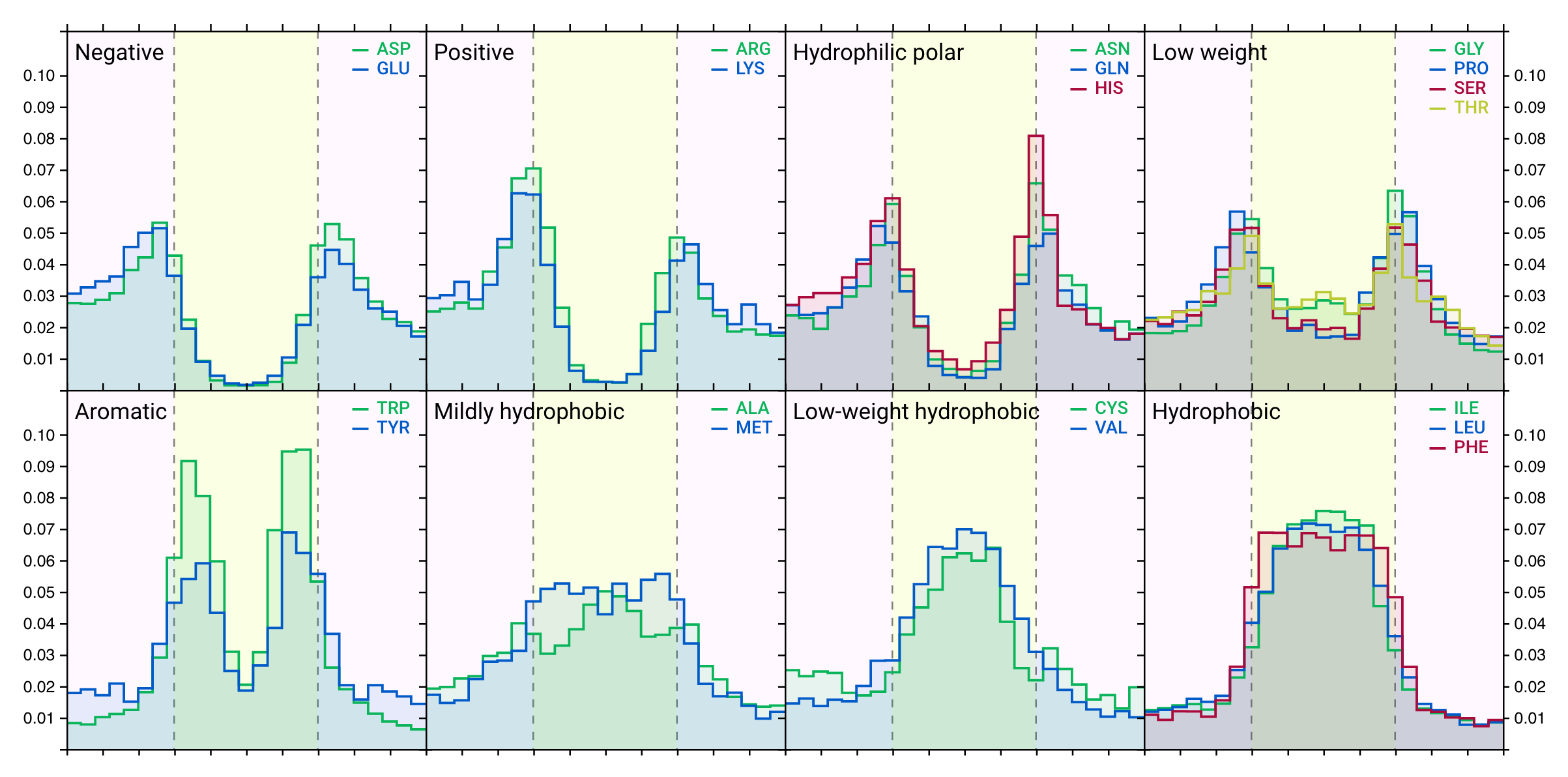
**

**Supplementary Figure 6: Histograms of amino acid frequency by adjusted displacement from bilayer centre.** Dashed vertical lines represent the positions of phosphate head-groups, which varies between simulations. The TM region is indicated in yellow, whilst extramembrane regions are shown in pink.


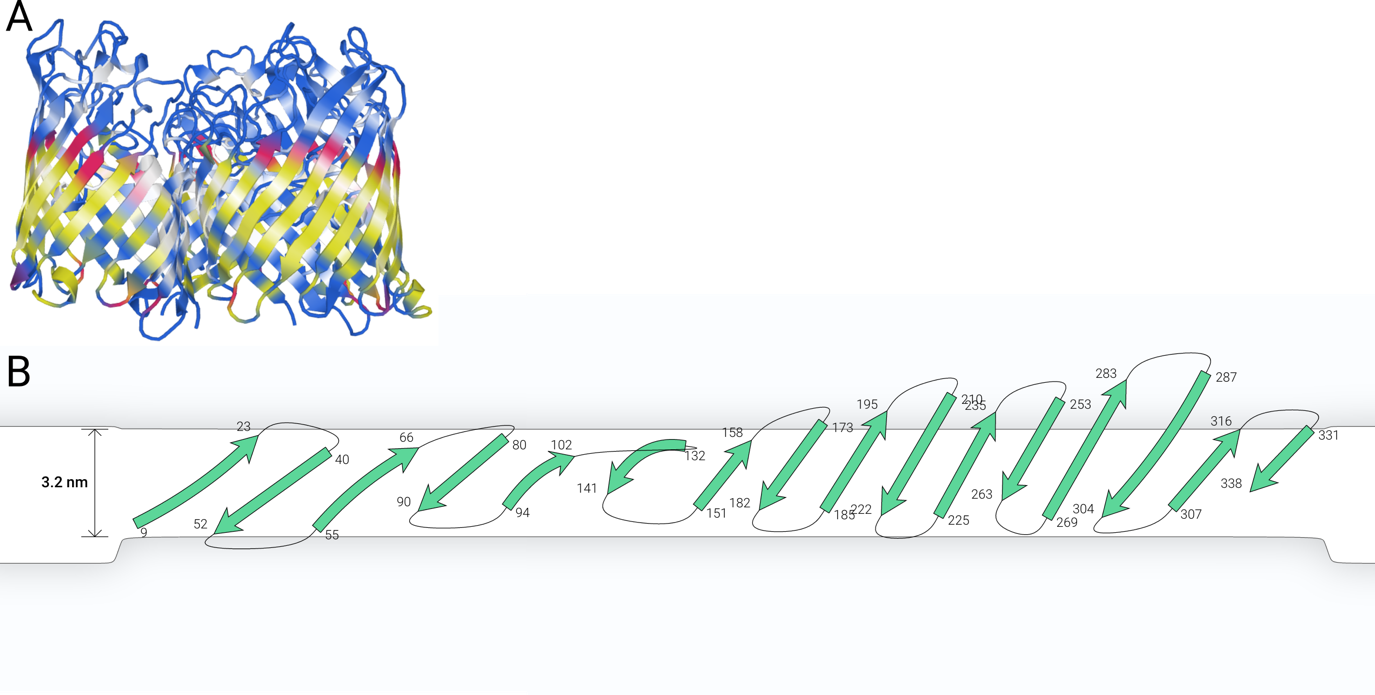


C


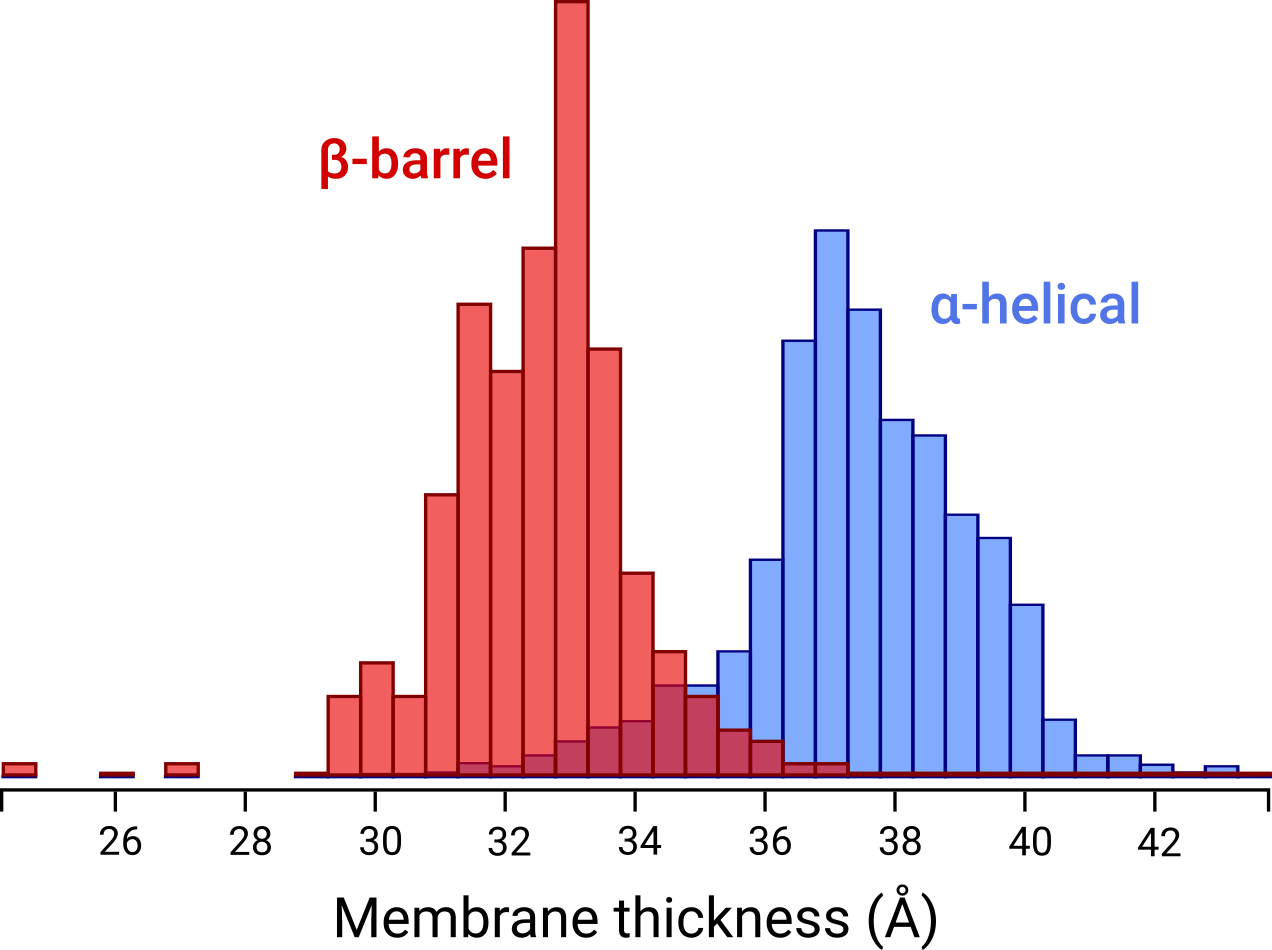


**Supplementary Figure 7: Structure, topology and membrane Interactions for** β**-barrel proteins.** A) OmpF porin (PDB id: 3K19) with coloured contacts for lipid headgroup (red) and tail (yellow) interactions. B) β-strand topology plot, showing the membrane thinning induced by the protein structure. C) Annular membrane thickness for β-barrel proteins (red) and α-helical proteins (blue). Thickness is calculated according to mean distance between phosphate beads of each leaflet in the first annular shell of lipids.
